# High levels of PF4, VEGF-A, and classical monocytes correlate with the platelets count and inflammation during active tuberculosis

**DOI:** 10.3389/fimmu.2022.1016472

**Published:** 2022-10-17

**Authors:** Alexia Urbán-Solano, Julio Flores-Gonzalez, Alfredo Cruz-Lagunas, Gloria Pérez-Rubio, Ivette Buendia-Roldan, Lucero A. Ramón-Luing, Leslie Chavez-Galan

**Affiliations:** ^1^ Laboratory of Integrative Immunology, Instituto Nacional de Enfermedades Respiratorias Ismael Cosío Villegas, Mexico City, Mexico; ^2^ Laboratory of Immunobiology and Genetic, Instituto Nacional de Enfermedades Respiratorias Ismael Cosío Villegas, Mexico City, Mexico; ^3^ HLA Laboratory, Instituto Nacional de Enfermedades Respiratorias Ismael Cosío Villegas, Mexico City, Mexico; ^4^ Translational Research Laboratory on Aging and Pulmonary Fibrosis, Instituto Nacional de Enfermedades Respiratorias Ismael Cosío Villegas, Mexico City, Mexico

**Keywords:** platelet, tuberculosis, classical monocytes, inflammation, pulmonary fibrosis

## Abstract

Platelets play a major role in coagulation and hemostasis; evidence supports the hypothesis that they also contribute to immunological processes. Increased platelet counts have been associated with poor prognosis in tuberculosis (TB). Platelet–monocyte aggregates have been reported in patients with TB, but it is still unclear if only one monocyte subpopulation is correlated to the platelet count; moreover, the platelet–monocyte axis has not been studied during latent tuberculosis (LTB). In this study, mononuclear cells and plasma were obtained from patients diagnosed with active drug-sensitive TB (DS-TB, n = 10) and LTB (n = 10); cytokines and growth factors levels associated to platelets were evaluated, and correlations with monocyte subpopulations were performed to identify a relationship between them, as well as an association with the degree of lung damage. Our data showed that, compared to LTB, DS-TB patients had an increased frequency of platelets, monocytes, and neutrophils. Although DS-TB patients showed no significant difference in the frequency of classical and non-classical monocytes, the classical monocytes had increased CD14 intensity of expression and frequency of TLR-2+. Furthermore, the plasma levels of angiogenic factors such as vascular endothelial growth factor (VEGF-A), platelet-derived growth factor (PDGF-BB), and platelet factor-4 (PF4), and pro-inflammatory cytokines like interleukin 6 (IL-6), interleukin 1 beta (IL-1β), and interferon-γ-inducible protein 10 (IP-10) were increased in DS-TB patients. In addition, PF-4 and VEGF-A correlated positively with the frequency of classical monocytes and the platelet count. Using a principal component analysis, we identified four groups of DS-TB patients according to their levels of pro-inflammatory cytokines, angiogenic factors, and degree of lung damage. This study establishes that there is a correlation between VEGF-A and PF4 with platelets and classical monocytes during active TB, suggesting that those cell subpopulations are the major contributors of these molecules, and together, they control the severity of lung damage by amplification of the inflammatory environment.

## Introduction

Tuberculosis (TB) is an infectious disease caused by *Mycobacterium tuberculosis* (Mtb) and is the second leading infection killer after the current COVID-19 pandemic. The World Health Organization (WHO) estimates 10 million new TB cases in 2020 (1). After being exposed to Mtb, 5%–10% of infected individuals develop active TB (ATB), and 90% develop a TB status called latent tuberculosis (LTB), where the patient controls the infection without eliminating Mtb. Approximately 10% of LTB patients will reactivate Mtb during their life due to failures in their immune system (2, 3). During LTB, the immune response maintains Mtb in a quiescent metabolic state without evidence of abnormalities in the clinical or radiological status of the patient (4). In contrast, during ATB, the replicating Mtb triggers an important inflammatory state that induces damage to the infection site, leading to clinical and radiological manifestations (5).

Platelets are well known for their role in coagulation and hemostasis. However, current evidence supports the hypothesis that they also contribute to diverse immunological processes (6). It has been suggested that platelets drive TB immunopathology through their effect on other immune cells (7). It was recently reported that they accumulate in the lung lesions of TB patients, influencing the activation and differentiation of macrophages (8, 9).

Evidence supports the adverse effects on the immune cells by platelets during Mtb infection; for example, a high platelet count has been associated with a poor prognosis during TB because there is tissue degradation; even antiplatelet agents have been proposed as an adjunct to antibiotic treatment (10). In addition, a report suggested that platelets restricted the oxidative burst favoring disease progression; Mtb-infected and platelet-depleted mice increased cellular reactive oxygen species (ROS) levels and reduced bacterial burden (11).

Platelet factor-4 (PF4) is produced and released specifically by platelets, and it has been found to increase in TB patients, correlating with clinical and radiological severity (12). In addition, other non-specific proteins to platelets, such as platelet-derived growth factor (PDGF-BB), regulated upon activation, normal T cell expressed and presumably secreted (RANTES), macrophage inflammatory protein 1 alpha (MIP1α), transforming growth factor-beta (TGF-beta), pentraxin 3, interleukin-1-beta (IL-1β) and vascular endothelial growth factor (VEGF-A) have been found increased in plasma from TB patients (8, 9, 13).

Mtb induces the secretion of VEGF-A by infected macrophages, favoring Mtb dissemination to extrapulmonary sites through newly formed blood vessels (14). VEGF-A also regulates the expression of matrix metalloproteinases (MMPs), leading to tissue destruction. MMP expression promotes cavitations and fibrosis in the lung (15–17). Moreover, VEGF-A promotes non-vascular functions; for instance, chemotaxis of monocytes, granuloma formation, and maintaining a pro-inflammatory environment (18). The relevance of using VEGF-A inhibitors as a host-directed therapy remains to be discussed in TB treatment (19).

Platelets mediate the recruitment of monocytes and macrophages through the release of PF4, which induces the migration of C–C chemokine receptor type 1 (CCR1)-expressing cells. CCR1 promotes inflammation because it regulates the traffic of immune effector cells when binding to its ligands (20, 21); this pathway suggests a link between an inflammatory environment and PF4 (22). Currently, it is uncertain how platelet activation impacts monocyte activation and inflammation status during LTB and ATB. Therefore, understanding the dynamics between platelets and monocytes is essential to clarifying the role of the platelets in maintaining intercellular communication that regulates inflammation. Here, we measured inflammatory cytokines and angiogenic factors in the plasma of LTB and ATB patients, and correlations with platelet count and monocyte subpopulation were established.

This study suggests that platelets may promote a hyperinflammatory environment through stimulation of classical monocytes, which in turn produce cytokines and factors to favor the activation and further production of platelets, a process associated with increased severity of lung damage and probable development of pulmonary fibrosis.

## Materials and methods

### Ethics statement

This study was approved by the Ethics Committee of the Instituto Nacional de Enfermedades Respiratorias Ismael Cosio Villegas (Code numbers B24-16, B07-18, and B06-22). All procedures performed in the study were conducted following the principles stipulated in the Helsinki Declaration, and all participants signed a written informed consent.

### Patients

**Figure 1:**
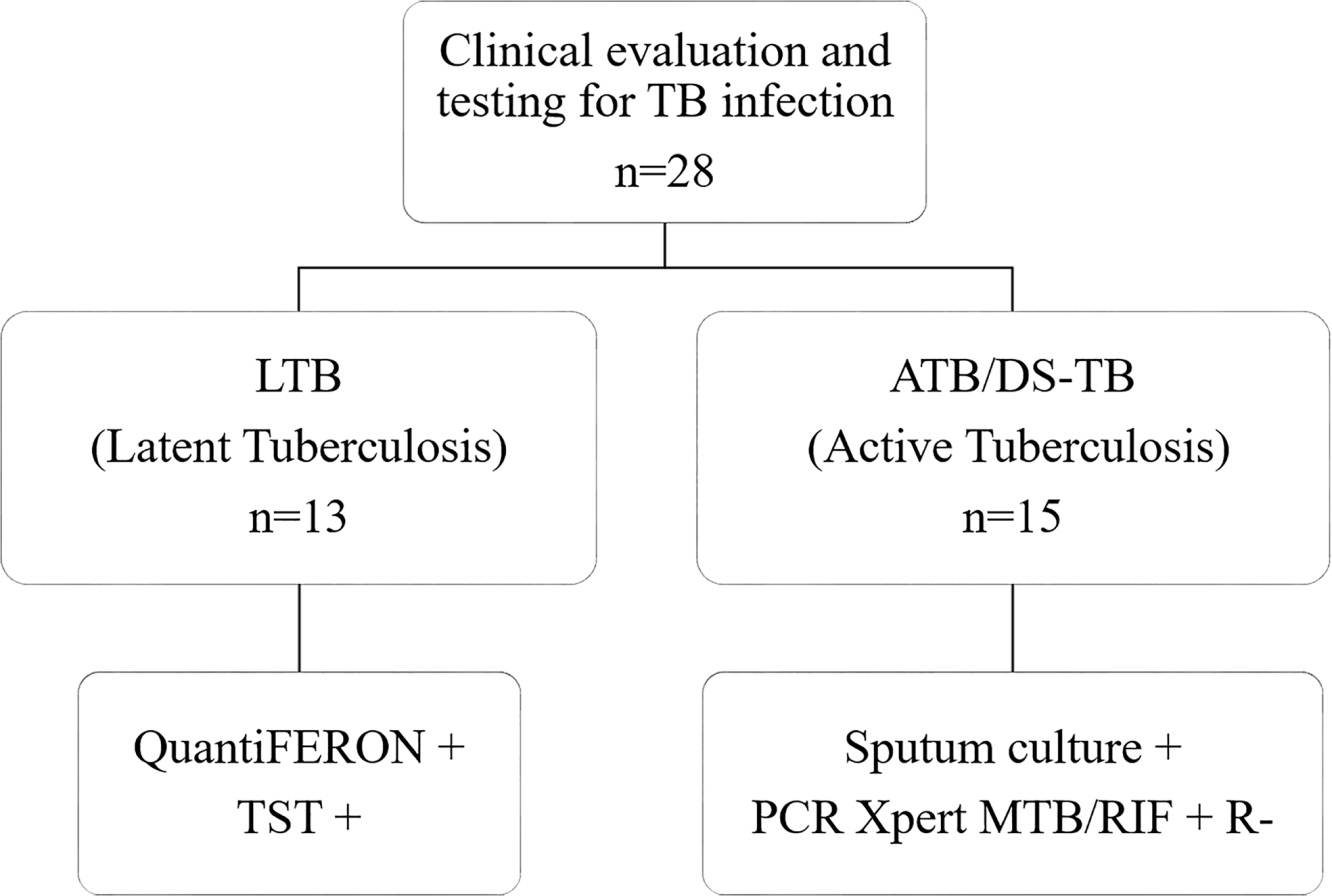
Workflow of enrolled patients. Twenty-eight subjects were recruited for this study; 13 were diagnosed with latent tuberculosis (LTB), and 15 with active drug-sensitive tuberculosis (DS-TB). TST, Tuberculin skin test; PCR Xpert MTB/RIF, Polymerase Chain Reaction Xpert *Mycobacterium Tuberculosis*/Rifampicin; R, resistant.

Twenty-eight patients >18-year-old with Mtb infection were enrolled during 2016–2018 at the Instituto Nacional de Enfermedades Respiratorias, Mexico City. Study populations were divided into two groups: patients with LTB (n = 13) with a positive QuantiFERON-Gold In-Tube test and a positive tuberculin skin test, and patients with ATB (n = 15) whose diagnosis was made by a clinician evaluation, positive sputum culture, and PCR Xpert MTB/RIF (**Figure 1**). All ATB patients had drug-sensitive tuberculosis (DS-TB). Patients with HIV infection, cancer, and those undergoing immunosuppressive or anticoagulant therapy were excluded. After the diagnosis, patients received the corresponding treatment following the international normative and a clinical follow-up. Clinical, laboratory, and radiological data were obtained from the medical records of all ATB patients.

### Blood samples

Circulating blood samples were obtained at diagnosis time before the initiation of anti-TB therapy. Plasma and peripheral blood mononuclear cells (PBMCs) were recovered using BD Vacutainer tubes (BD Biosciences, San Jose, CA, USA). PBMCs were isolated by standard LymphoprepTM (Accurate Chemical-Scientific, Westbury, NY, USA) centrifugation gradient within 1 h of the blood draw and were subsequently cryopreserved. Plasma was obtained and stored at −70°C until use.

### Flow cytometry analysis

PBMCs were prepared to evaluate cell surface marker expressions using monoclonal antibodies (mAbs) against CD14, CD16, human leukocyte antigen-DR (HLA-DR), integrin alpha M subunit (CD11b), toll-like receptor-2 (TLR-2), TLR-4, and L-selectin (CD62-L). All the mAbs were provided by BioLegend (San Diego, CA, USA).

The cells used for the Fluorescence Minus One (FMO) condition were stained and acquired in parallel to identify background levels of staining; dead cells were discarded using viability staining Zombie Red Dye solution (BioLegend). More details of the antibodies used can be found in **Table S1**. The data were acquired using a FACS Aria II flow cytometer (BD Biosciences, San Jose, CA, USA) equipped with the FACSDiva 6.1.3 software (BD Biosciences, San Jose, CA, USA). In each condition, at least 50,000 events were acquired per sample. The flow cytometry data file (FCS) was analyzed using Flow Jo (Flow Jo, LLC, Ashland, OR, USA)™ v10.6.1.

The frequency (percentage) and the mean fluorescence intensity (MFI) were obtained for all molecules. Briefly, the analysis strategy consisted of limiting the singlet cells through forward scatter (FSC-A versus FSC-H), and viability plots were selected (Zombie Red negative, **Figure S1**).

### Soluble molecules evaluation

A Bio-plex Pro Human Cytokine Custom Panel (Bio-Rad Laboratories, Hercules, CA, USA) was used to evaluate the plasma levels of IL-6, IL-1β, interferon gamma-induced protein 10 (IP-10), PDGF-BB, and VEGF-A, following the instructions of the manufacturer. The data were acquired through a Bio-Plex 200 System and analyzed using Bio-Plex Manager 6.1 software (Bio-Rad Laboratories, Hercules, CA, USA).

Levels of platelet factor 4 (PF4/CXCL4) (R&D Systems, Minneapolis, MN, USA), monocyte chemoattractant protein-1 (MCP-1) (BioLegend, San Diego, CA, USA) and mucin 5 subtype B (MUC5B) (MyBioSource, San Diego, CA, USA) were measured in plasma using a sandwich-type Enzyme-Linked Immunosorbent Assay (ELISA) according to the instructions of the manufacturer. More details of the Bio-Plex and ELISA kits used are in **Table S1**. All proteins were quantified by comparison with the corresponding standard curve, and the optical density was measured using a microplate reader (Imark, Bio-Rad, Hercules, CA, USA).

Plasma from healthy donors (n = 10) was used to obtain the reference values for soluble molecule quantification (**Table S2**).

### Statistical analysis

Data are shown as median values and interquartile ranges (IQR, 25–75). For comparisons between groups, Mann–Whitney tests were used for comparisons between groups. Spearman one-tailed correlations were performed between clinical data, soluble molecule values, and phenotypic characterization data by flow cytometry of patients with latent tuberculosis (n = 7) and active tuberculosis (n = 5). Values of *p <*0.05 were considered statistically significant. Statistical analysis was performed using GraphPad Prism V 9.0.2 (GraphPad Software, Inc., San Diego, CA, USA).

Principal component analysis (PCA) was performed to reduce dataset dimensionality and detect patterns of the soluble molecules measured in TB patients, using GraphPad Prism, treating each patient as one data point. We observed and related specific points that appeared in relation to the others along the two selected components to infer clusters from PCA data.

Pairwise correlations were calculated and visualized as a correlogram using R studio version 1.4.1106 with the following libraries: ggplot2, ggcorrplot, corrplot, and tidyverse. Spearman’s correlation coefficient was indicated by square and heat scale; significance was indicated by *p <0.05, **p <0.01, and ***p <0.001.

### Protein network analysis

We used a computational prediction using STRING (Search Tool for the Retrieval of Interacting Genes/Proteins) to construct a protein–protein interaction network, indicating functional and physical protein associations. The STRING resource is available online at https://string-db.org/. We were set to visualize only the “evidence” interactions, with a maximum of 10 interactors for the first shell and five for the second shell, and a high score (0.7) as a minimum required interaction score. STRING analysis integrates all known and predicted associations between proteins, including physical interactions and functional associations. Functional enrichments in the protein network were supported by the REVIGO analysis as a well-known classification system based on Gene Ontology. Finally, to establish a powerful visual map across these data, we performed an advanced analysis and modeling using Cytoscape V 3.9.1, selecting the first neighbors to PF4 and VEGF-A nodes and showing them in a circular layout. Ultimately, we analyzed molecules shared between nodes using a Veen and Euler diagram app.

## Results

### Characteristics of the study population

The demographic characteristics of LTB and DS-TB patients are summarized in **Table 1**. The median age was 49 years for LTB and 38 for DS-TB, and female sex was predominant for both groups. DS-TB patients had a lower BMI value than LTB [LTB 31 (23–30) vs DS-TB 21 (18–22, 31, 32), *p <*0.0001]. The comorbidity of diabetes mellitus type 2 was present in 40% of DS-TB patients and 8% of LTB patients. A total of 100% of DS-TB and 85% of LTB patients received the Bacillus Calmette–Guérin (BCG) vaccine during childhood.

**Table 1:** Participant characteristics.

	LTB (n = 13)	DS-TB (n = 15)	*p*
Age (years)	49 (31–58)	38 (30–44)	ns
Male, n (%)	3 (23)	5 (33)	ns
Female, n (%)	10 (77)	10 (67)	ns
Body Mass Index (kg/m^2^)	31 (27–34)	21 (18–24)	****
Diabetes Mellitus type 2, n (%)	1 (8)	6 (40)	ns
Arterial hypertension, n (%)	1 (8)	1 (7)	ns
Previous pulmonary disease^†^, n (%)	0	1 (7)	ns
Smoking, n (%)	1 (8)	3 (20)	ns
BCG vaccination, n (%)	11 (85)	15 (100)	ns

Data is represented with median and interquartile range. The statistical comparison was performed using the Mann–Whitney U Test (****p <0.0001, ns, not significant), †previous diagnosis of Chronic Obstructive Pulmonary Disease (COPD) and asthma.

DS-TB patient data showed important changes in the hematological parameters compared to LTB. DS-TB patients had a higher absolute count of circulating total leukocytes than LTB (*p <*0.05). The counts of neutrophils (*p <*0.001) and monocytes (*p <*0.001) were increased, whereas lymphocytes were decreased (*p <*0.001). Moreover, DS-TB displayed a higher platelet count than LTB (*p <*0.0001), and in line with previous reports, hemoglobin and hematocrit were lower in DS-TB than in LTB (p <0.001 for both) (**Table 2**).

**Table 2:** Laboratory data of hematological parameters.

	LTB	DS-TB	*p*
Leukocytes (RV 4.4–11.3 × 10^3^ cells/mm^3^)	7 (6–8)	8 (7–11)	*
Absolute neutrophil count (RV 1.7–7.6 × 10^3^ cells/mm^3^)	4 (3–5)	6 (5–8)	***
Neutrophil percentage (RV 55–62%)	59 (54–65)	70 (66–82)	**
Absolute monocyte count (RV 0.3–0.9 ×10^3^ cells/mm^3^)	0.4 (0.4–0.5)	0.6 (0.6–0.9)	***
Monocyte percentage (RV 4%–10%)	6 (5–8)	9 (6–11)	*
Absolute lymphocyte count (1.0–3.2 × 10^3^ cells/mm^3^)	2 (2–3)	1 (1–2)	*
Lymphocyte percentage (20%–40%)	31 (28–36)	18 (10–22)	***
Platelets (RV 150–400 × 10^3^ cells/mm^3^)	223 (198–254)	433 (317–507)	****
Hemoglobin (RV 15–18.5 g/dl)	15 (14–16)	13 (11–14)	***
Hematocrit (RV 45%–53%)	45 (43–50)	38 (34–43)	***

Data is represented with median and interquartile range. The statistical comparison was performed using Mann–Whitney U Test (*p <0.05, **p <0.01, ***p <0.001, ****p <0.0001). RV, Reference value (provided by the institutional Clinical Laboratory).

Finally, biochemical parameters showed that compared to LTB, DS-TB patients had lower levels of albumin (*p* = 0.0046), total bilirubin (*p* = 0.0475), alanine transaminase (*p* = 0.0422), and creatine phosphokinase (*p <*0.0001) (**Table 3**).

**Table 3:** Laboratory data of biochemical parameters.

	LTB	DS-TB	*p*
Glucose (RV 70–99 mg/dl)	103 (95–111)	98 (94–156)	ns
Creatinine (RV 0.8–1.3 mg/dl)	0.73 (0.6–0.9)	0.67 (0.5–0.7)	ns
Urea (RV 15 – 58 mg/dl)	21 (18–32)	22 (16–26)	ns
Blood urea nitrogen (RV 8–27 mg/dl)	10 (9–15)	10 (8–12)	ns
Uric Acid (RV 3.5–7.2 mg/dl)	5 (5–7)	5 (4–6)	ns
Total Protein (RV 6.5–8.1 g/dl)	7 (7–8)	8 (7–8)	ns
Albumin (RV 3.5–5.0 g/dl)	4 (4–4)	3 (2–4)	**
Total bilirubin (RV 0.2–1.0 mg/dl)	0.66 (0.5–1)	0.55 (0.4–0.6)	*
Aspartate transaminase (RV 12–35 U/L)	24 (20–28)	26 (18–32)	ns
Alanine transaminase (RV 9–47 U/L)	21 (15–30)	16 (12–17)	*
Lactate dehydrogenase (RV 139–205 U/L)	139 (131–171)	166 (140–233)	ns
Alkaline phosphatase (RV 40–129 U/L)	77 (68–104)	90 (75–114)	ns
Creatine phosphokinase (RV 30–223 U/L)	93 (70–136)	34 (27–44)	****
Glycated hemoglobin (RV <5.7%)	6 (5–6)	6 (6–9)	ns

Data is represented with median and interquartile range. The statistical comparison was performed using Mann–Whitney U Test (*p <0.05, **p <0.01, ****p <0.0001, ns, not significant). RV, Reference value (provided by the institutional Clinical Laboratory).

In summary, DS-TB patients presented co-morbidities and lower BMI compared to LTB. Notably, the DS-TB showed high levels of platelets and immune cell subpopulations, except for lymphocytes, and a profile related to anemia.

### Classical monocytes from DS-TB patients have increased intensity of expression of CD14 and CD11b

**Figure 2:**
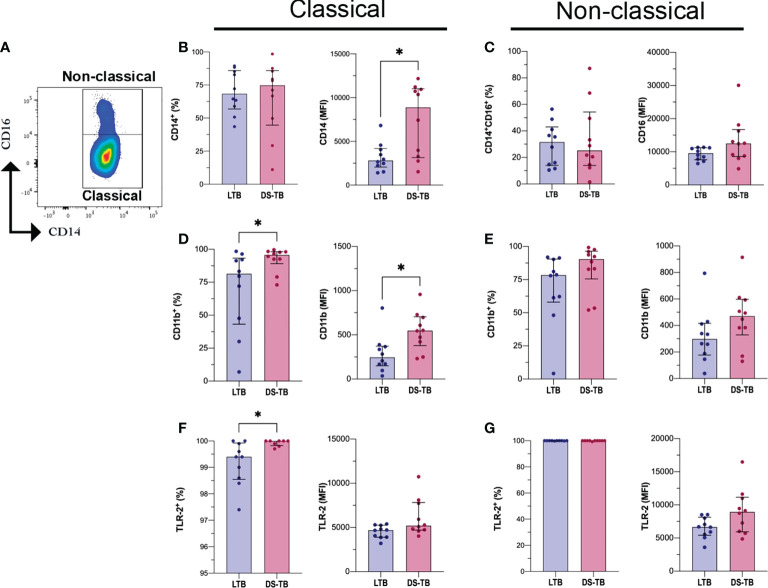
DS-TB patients have classical monocytes with CD14, CD11b and TLR-2 altered expression. **(A)** Identification of monocytes subpopulations based on CD14 and CD16 expression as Classical (CD14^+^) and Non-classical (CD14^+^CD16^+^). **(B)** Frequency and mean fluorescent intensity (MFI) of CD14 on classical monocytes, comparison between latent and active tuberculosis patients. **(C)** Frequency and MFI of CD16 on non-classical monocytes, comparison between latent and active tuberculosis patients. Frequency and MFI of CD11b and TLR-2 on classical monocytes (**D, F**, respectively) and non-classical monocytes (**E, G**, respectively). Data are shown as median with interquartile range (IQR, 25–75). The statistical comparison was performed using Mann–Whitney U Test (*p <0.05), n = 10 for both groups.

Monocytes are a precursor population to macrophages, one of the most important cell subpopulations during the immune response against Mtb (31). Having identified that DS-TB patients have an increased monocyte count compared to LTB (**Table 2**), the next aim was to verify if the frequencies of classical (CD14+CD16−) and non-classical (CD14+CD16+) monocytes were different between LTB and DS-TB, and this was evaluated by flow cytometry (**Figure 2A**).

We did not find differences in the frequency of monocyte subsets between TB patient groups (**Figures 2B**, left, **C**, left). However, the CD14 intensity of expression (reported as MFI) was increased in classical monocytes of DS-TB patients compared to the LTB group [8,889 (3,140–11,009) vs 2,817 (2,066–4,192) MFI, respectively, *p* = 0.0232] (**Figure 2B**, right), while CD16 intensity of expression was not modified on non-classical monocytes (**Figure 2C** right).

On both classical and non-classical monocytes, human leukocyte antigen–DR isotype (HLA-DR), integrin alpha-M (CD11b), Toll-like receptor 2 (TLR-2), and toll-like receptor 4 (TLR-4) expression were evaluated. The classical monocytes from DS-TB patients, compared with the LTB group, displayed a higher frequency of CD11b+ [95.65 (89–98) vs 81.45 (43–93), respectively, *p* = 0.0355] and CD11b MFI [548 (DS-TB: 379–704) vs LTB: 244 (150–371), *p* = 0.0115] (**Figure 2D**). Regarding non-classical monocytes, we did not observe differences (**Figure 2E**). Moreover, the classical monocytes from DS-TB, compared to LTB, had an increased frequency of TLR-2+ [100 (99.8–100) vs 99.4 (98.5–99.9), respectively, *p* = 0.0139] but not the TLR-2 MFI (**Figure 2F**). TLR2 expression in non-classical monocytes showed no difference between groups (**Figure 2G**). Finally, the frequency and MFI of HLA-DR, TLR-4, and CD62L were not different between the groups (**Figure S2**).

These results suggest that mainly classical monocytes are affected during DS-TB compared to LTB. CD11b and TLR-2 may play a role in favoring the activation of classical monocytes, probably to maintain inter-cellular communication through cytokine production.

### DS-TB patients have increased plasma levels of PF4 and VEGF-A

**Figure 3:**
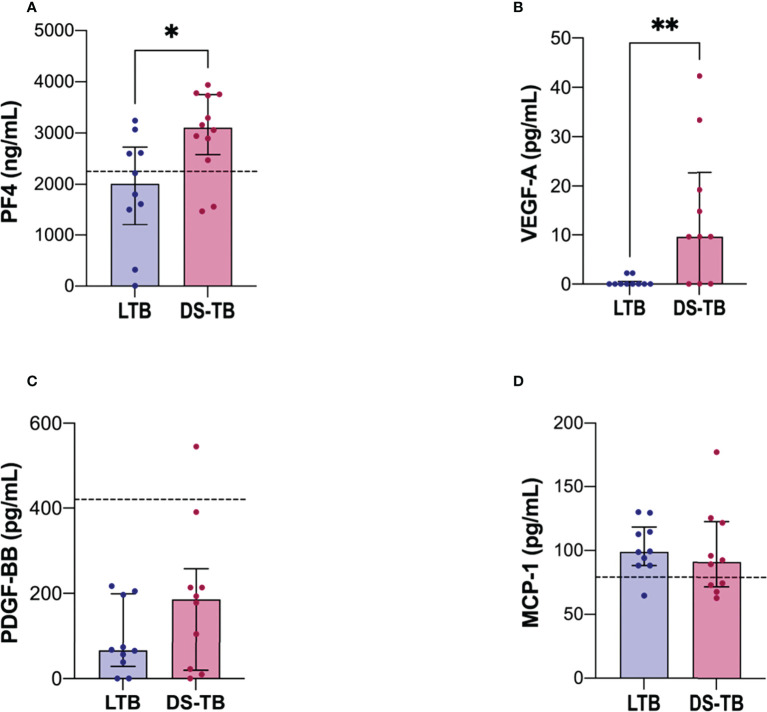
DS-TB patients have increased the soluble levels of molecules associated with a platelet activation profile. Soluble molecules evaluated in plasma: **(A)** PF4, **(B)** VEGF-A, **(C)** PDGF-BB, and **(D)** MCP-1. The black dashed lines represent the median of healthy donors (for VEGF-A, they had indetectable values, not visible). Data are shown as median with interquartile range (IQR, 25–75). The statistical comparison was performed using Mann–Whitney U Test (*p <0.05, **p <0.01), n = 10 for both groups, PF4 and MCP-1 had n = 8 for both groups. PF4, platelet factor-4; PDGF-BB, platelet-derived growth factor; VEGF-A, vascular endothelial growth factor; MCP-1, monocyte chemoattractant protein-1.

Because our data showed that the platelet count is increased in DS-TB patients (**Table 2**), plasma levels of angiogenic-related factors such as PF4, PDGF-BB, VEGF-A, and MCP-1 were evaluated to assess if platelets were activated. Higher levels of PF4 were identified in the DS-TB group than in the LTB group [LTB 2,012 (1213–2727) vs DS-TB 3,108 (2,578–3,750), *p* = 0.0249] (**Figure 3A**). Similar data were observed with VEGF-A [LTB 0 (0–0.55) vs DS-TB 9.64 (0–22.69), *p* = 0.0067] (**Figure 3B**). PDGF-BB and MCP-1 levels did not show changes (**Figures 3C, D**).

Thus, our results suggest that the high amounts of PF4 exhibited by DS-TB patients were delivered by activated platelets, whereas the VEGF-A levels could be produced by both platelets and monocytes.

### DS-TB patients have increased levels of IL-6, IL1-β and IP-10

**Figure 4:**
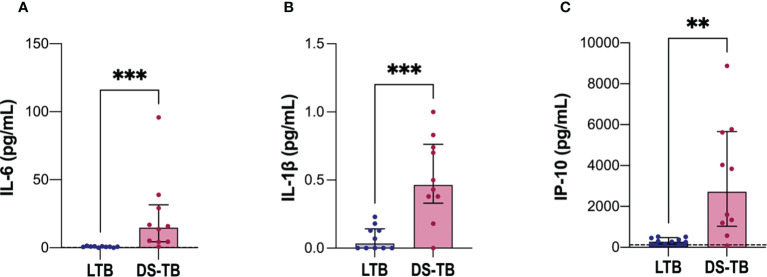
Increased soluble levels of pro-inflammatory cytokines in patients with DS-TB. Cytokines evaluated in plasma: **(A)** IL-6, **(B)** IL-1β, and **(C)** IP-10. The black dashed lines represent the median value reported of healthy donors for IP-10 (for IL-6, the median is equal to 0.22 pg/ml, and for IL-1β they had indetectable values, not visible). Data are shown as median with interquartile range (IQR, 25–75). The statistical comparison was performed using Mann–Whitney U Test (**p <0.01, ***p <0.001), n = 10 for both groups IL-6, interleukin-6; IL-1β, interleukin-1 beta; IP-10, Interferon-γ inducible protein 10.

To evaluate the inflammatory status according to the clinical form of TB, we evaluated inflammatory cytokines in plasma of LTB and DS-TB. IL-6 levels [LTB 0.73 (0.4–1.0) vs DS-TB 15 (4–32), *p* = 0.0002] (**Figure 4A**), IL-1β [LTB 0.035 (0–0.14) vs DS-TB 0.465 (0.33–0.76), *p* = 0.0009) (**Figure 4B**) and IP-10 [LTB 282 (162–474) vs DS-TB 2,718 (1,033–5,658), *p* = 0.0015) (**Figure 4C**) were increased in DS-TB patients compared to LTB. These results showed that DS-TB patients display a systemic pro-inflammatory status.

### DS-TB patients show strongly positive correlations between classical monocyte–platelet–platelet factors

Platelets stimulate monocyte chemotaxis, but currently, it is unclear if, specifically, only one monocyte subpopulation is related to platelets or factors delivered by platelets, such as PF4 and VEGF-A. Therefore, we performed correlograms to determine if there is a link between platelet–monocyte-inflammation.

**Figure 5:**
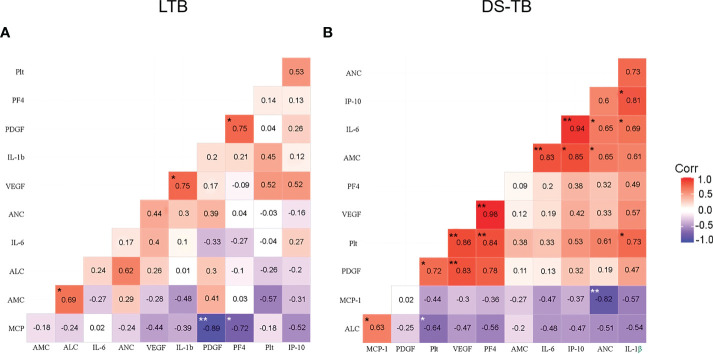
Correlation between blood cell count, platelet factors and pro-inflammatory cytokines in latent **(A)** and active **(B)** TB patients. Correlations are presented with the value of Spearman’s Rho in the corresponding box. Significant correlations are represented with asterisks (*p <0.05, **p <0.01, white asterisk for negative correlation, and black asterisk for positive correlation). The red color indicates a strong positive correlation, and the blue indicates a strong negative correlation.

First, we evaluated if the absolute monocyte count (AMC) correlates with the absolute neutrophil count (ANC), absolute lymphocyte count (ALC), platelet count (Plt), pro-inflammatory cytokines, and platelet factors (**Figure 5**). LTB showed a positive correlation between AMC and ALC (p*, rho = 0.69), VEGF-A with IL1β (p*, rho = 0.75), and PF4 with PDGF-BB (p*, rho = 0.75); LTB did not show significant correlations between platelets count and factors produced by them (**Figure 5A**). On the other hand, DS-TB displayed strong positive correlations with PF4 (p**, rho = 0.84), VEGF-A (p**, rho = 0.86), and PDGF-BB (p*, rho = 0.72) with total platelet count (Plt). In the same way, VEGF-A correlated positively with PDGF-BB (p**, rho = 0.83) and with PF4 (p**, rho = 0.98). IL-6 (p**, rho = 0.83), and IP-10 (p*, rho = 0.85) were positively correlated with AMC; IL-6 (p*, rho = 0.65) also showed a positive correlation with the absolute neutrophil count (ANC). On the contrary, MCP-1 (p**, rho = −0.82) had a negative correlation with ANC.

Posteriorly, we performed correlation analysis with platelets using the total and monocyte subsets data obtained by flow cytometry. LTB patients showed some positive correlations of molecules expressed on the total monocytes (TM) [VEGF-A and IL1-β (p*, rho = 0.81), HLA-DR and TLR-2 (p*, rho = 0.79); HLA-DR and IL1-β (p*, rho = 0.78); and CD62L and TLR-2 (p*, rho = 0.82)] (**Figure S3A**). DS-TB showed a positive correlation between TM frequency and IL-1β (p**, rho = 1) and CD14 (p*, rho = 0.9), whereas VEGF-A and PF4 correlated positively between them (p**, rho = 0.97) (**Figure S3B**).

**Figure 6:**
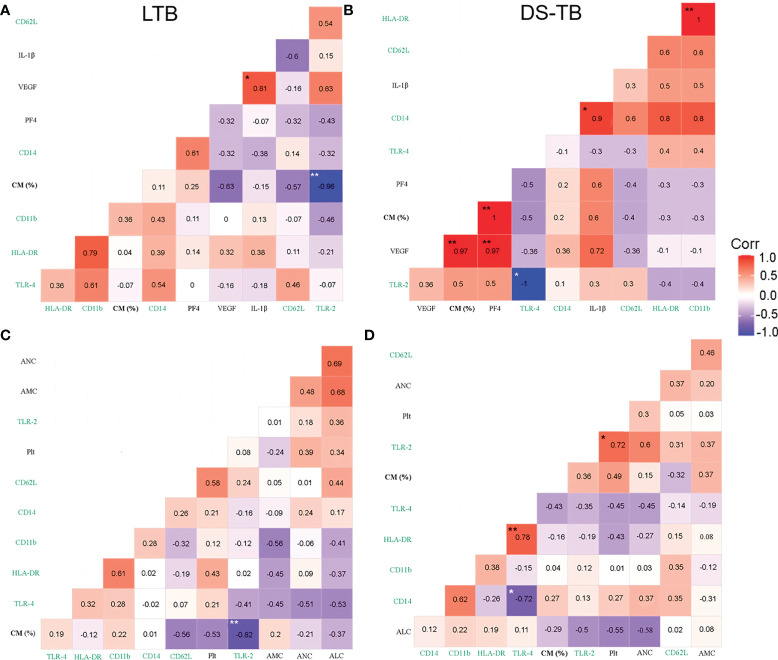
Correlation between blood cell count, platelet factors and pro-inflammatory cytokines with monocyte phenotypical characterization in TB patients. **(A, B)** Correlation between classical monocytes (CM) frequency, surface markers and plasmatic levels of soluble molecules in latent **(A)** and active **(B)** TB patients, n = 8 for both groups. **(C, D)** Correlations between CM frequency and blood cell count in latent **(C)** and active **(D)** TB patients, n = 10 for both groups. Correlations are presented with the value of Spearman’s Rho in the corresponding box. Significant correlations are represented with asterisks (*p <0.05, **p <0.01, white asterisk for negative correlation, and black asterisk for positive correlation). The red color indicates a strong positive correlation, and the blue indicates a strong negative correlation. Green labels are expressed in MFI.

LTB showed a negative correlation between the frequency of classical monocytes (CM) and TLR-2 (p**, rho = −0.96) (**Figures 6A, C**). DS-TB patients showed a strong positive correlation between CM and VEGF-A (p**, rho = 0.97), PF4 (p**, rho = 1) (**Figure 6B**), suggesting CM plays a role in inducing the secretion of these factors. Similarly, DS-TB patients had a positive correlation between CD14 MFI and IL-1β (p*, rho = 0.9), VEGF-A and PF4 (p**, rho = 0.97), and TLR-2 had a negative correlation with TLR-4 MFI (p*, rho = −1) (**Figure 6B**). CM from LTB did not correlate with other immune subpopulations; moreover, Plt did not correlate with TLR-2 or TLR-4 (**Figure 6C**). DS-TB had a positive correlation with Plt and TLR-2 (p*, rho = 0.72) (**Figure 6D**). It is essential to note that panels A and B had eight patients per group. In contrast, panels C and D had 10 patients per group. The discrepancy in sample size is because PF4 and MCP-1 were not measured in all patients. Although the correlograms showed slight differences in the rho value when comparing the same parameters, the behavior still had similar values and tendencies.

LTB patients had a positive correlation between non-classical monocytes (NCM) and TLR2 (p*, rho = 0.79) (**Figure S4A**), whereas DS-TB subjects had a negative correlation between NCM and VEGF-A (p**, rho = −0.97), and PF4 (p**, rho = −1) (**Figure S4B**).

Together, these correlations showed that in DS-TB, CM, probably through TLR-2, favored the pro-inflammatory microenvironment; moreover, Plt correlated positively with TLR-2, suggesting the presence of a monocyte–TLR2–platelet-inflammation axis.

### PCA reveals that DS-TB patients can be divided into four clusters

**Figure 7:**
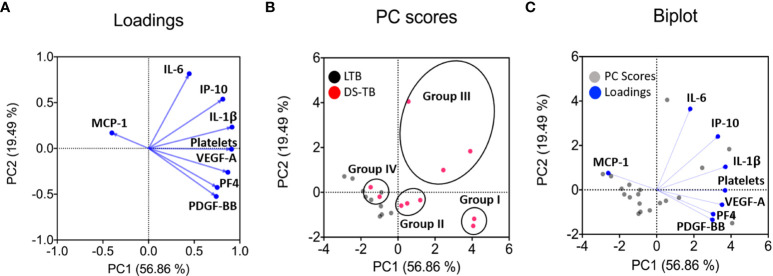
Principal component analysis (PCA) shows main dimensions of variation soluble molecules measured in patients diagnosed with TB. The PCA was constructed with eight clinical variables measurements [monocyte chemoattractant protein-1 (MCP-1), interleukin 6 (IL-6), interferon gamma-induced protein 10 (IP-10), interleukin 1 beta (IL-1β), platelets count, vascular endothelial growth factor (VEGF-A), platelet factor 4 (PF4) and platelet-derived growth factor (PDGF-BB). The correlation of each of these variables with the first two principal components is shown in a loadings plot **(A)**, demonstrating that the first principal component (PC1) was dominated by markers of systemic inflammation and angiogenic factors (platelets count, IL-1β, and VEGF-A were the most important). In contrast, the second principal component (PC2) was associated with IL-6. The PC scores plot showed that the data from 20 samples of people infected with TB were significant **(B)**. In the score plot, red and black dots represent a patient and is colored in accordance with the embedded legends. Finally, a biplot was used to show the loadings and PC scores **(C)**. These PCs explained 56.86% and 19.49% variation in the eight clinical variables measurements, respectively.

Dimension reduction by PCA was applied to LTB and DS-TB groups that were adjusted to score distance (SD) for the following variables: VEGF-A, PDGF-BB, IL-6, IP-10, PF4, IL-1β, MCP-1, and platelet count. In addition, we applied PCA to all individuals with the same parameters between groups to map differences between patients with DS-TB and LTB. The first principal component (PC1) summarized 56.86% of the variation captured by markers of systemic inflammation and angiogenic factors such as platelet count, IL-1β, and VEGF. The second principal component (PC2) summarized 19.49% of variation, and it had as its main variation IL-6, a marker of systemic inflammation (**Figure 7A**).

The PC score plot shows the analysis of samples from 20 patients, who had completed all the variables quantified to obtain PC1 and PC2; the LTB patients (black dot) could be easily distinguished from DS-TB (pink dot) (**Figures 7B, C**). On the contrary, DS-TB can be divided into IV groups according to PC1 and PC2 profiles (**Figure 7B**). More information on clinical and radiological data provided patient-by-patient is shown in **Table 4**. Group I included 2 DS-TB patients characterized by a high level of inflammatory and angiogenic factors and the presence of cavitations. Group II included three DS-TB patients, characterized by a high level of angiogenic factors but no inflammatory cytokines and lung affection with or without cavitations. Group III included three DS-TB patients, characterized by high levels of inflammatory cytokines but no angiogenic factors and destruction of lung parenchyma and cavitation. Group IV included two DS-TB patients, characterized by a low level of inflammatory and angiogenic factors (similarly to LTB); however, they showed lung affectation without cavitations.

**Table 4:** Identified groups of DS-TB patients and radiological characteristics.

	# patient	Radiological characteristics		Comorbidities	Average age (years)
**GROUP I.** **Pro-inflammatory cytokines and platelet factors elevation**	1	Two caverns in RLL	100% Presence of cavitations	50% Type 2 Diabetes mellitus	41.5
	2	Caverns in LUL			
**GROUP II.** **Platelet factors elevation but not pro-inflammatory cytokines**	3	Both upper lobes with cavitated lesions	66.6% presence of cavitations. 33.3% Lung affection without cavitations.	33.3% Type 2 Diabetes mellitus	39
	4	Left apical caverns, cavern at the level of lingula, superior segment of LLL with two caverns			
	5	Right basal consolidation, reticulonodular pattern			
**GROUP III.** **Main elevation of pro-inflammatory cytokines**	6	Adenopathies, multiple cavitations. Loss of left pulmonary parenchyma.	66.6% presence of adenopathies. 100% destruction of lung parenchyma. 100% presence of cavitations.	100% Type 2 Diabetes mellitus. 33.3% Previous pulmonary disease. 33.3% Dyslipidemia. 33.3% Smoking.	44
	7	Adenopathies, multiple cavitations, destruction of right lung parenchyma			
	8	Multiple caverns. Lung parenchymal destruction in the LUL.			
**GROUP IV. Low levels of platelet factors and pro-inflammatory cytokines**	9	Pulmonary nodule	100% Lung affection without cavitations	None	35.5
	10	Bilateral reticular pattern, pleural thickening			

RLL, Right Lower Lobe; LUL, Left Upper Lobe; LLL, Left Lower Lobe.

### DS-TB patients have increased MUC5B levels and lung damage

Our data suggested that patients with higher levels of pro-inflammatory cytokines and/or platelet factors also presented greater radiological severity. Mucin 5B (MUC5B) mediates the production of mucous; its overexpression leads to hyperproduction of mucus accumulated in the bronchoalveolar region and produces chronic inflammation. Moreover, it has been related to the development of idiopathic pulmonary fibrosis (IPF) (32, 33). Thus, we evaluated the MUC5B level to explore possible fibrotic lung damage in DS-TB patients.

**Figure 8:**
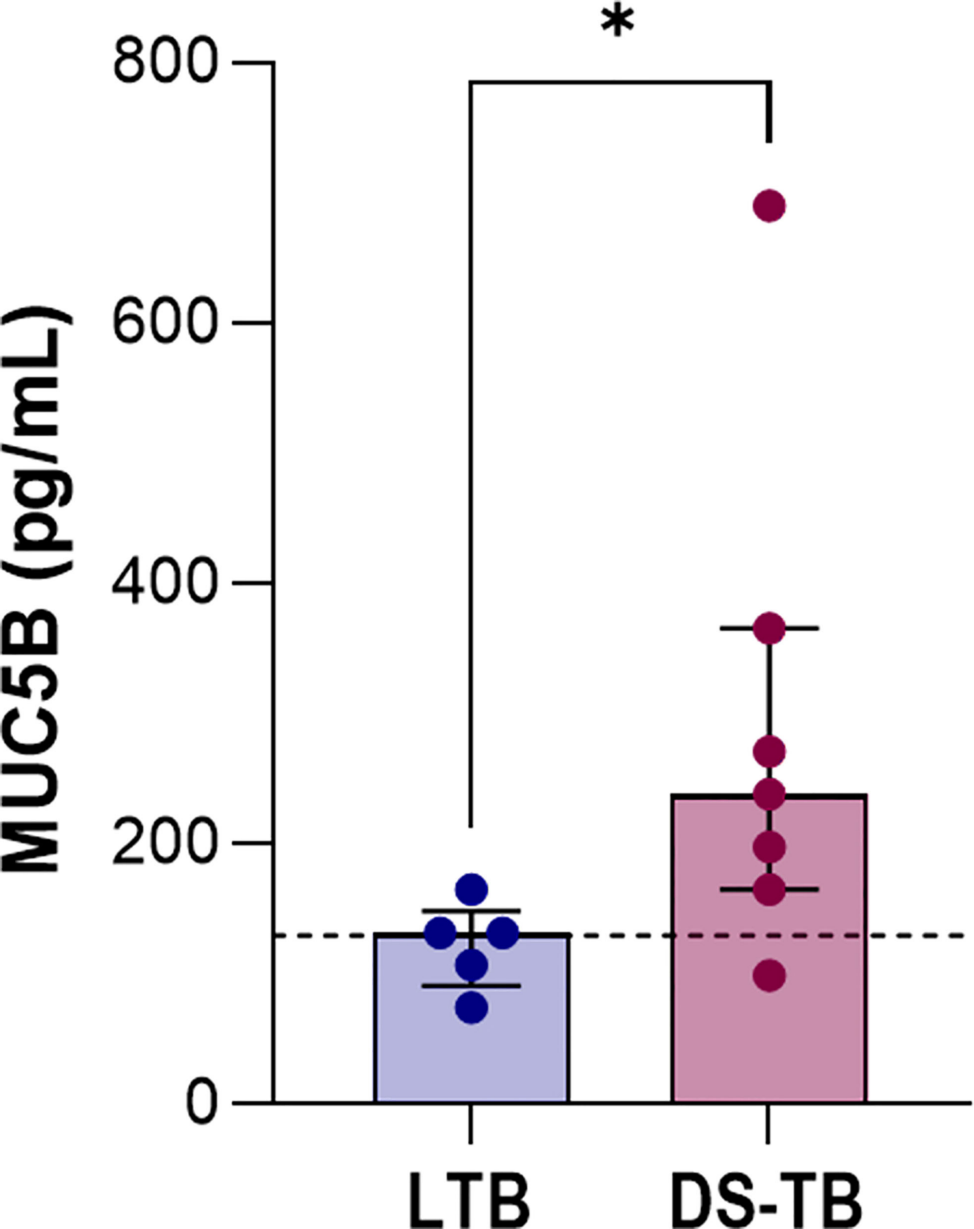
Increased levels of MUC5B in patients with active TB. Data are shown as median with interquartile range (IQR, 25–75). The black dashed lines represent the median value reported for healthy donors. The statistical comparison was performed using Mann–Whitney U Test (*p <0.05). n = 8 for both TB groups. MUC5B, mucin 5B.

DS-TB patients had higher MUC5B levels than LTB [LTB 131.9 (91–148) vs DS-TB 238.5 (165–365), *p* = 0.0427] (**Figure 8**), and we confirmed by chest X-ray data that MUC5B levels were associated with more significant pulmonary damage characterized by bigger cavitations and the destruction of pulmonary parenchyma. This result supports the hypothesis that the high presence of platelets could not benefit active TB patients; the platelet/monocyte axis probably induces high pulmonary destruction, characteristics that could be associated with a future fibrotic process.

### According to STRING analysis VEGF-A mediates inflammation and angiogenesis, whereas PF4 only inflammation in DS-TB patients

A STRING analysis was performed to predict possible interactions between PF4, VEGF-A, PDGF-BB, IL-6, IP-10, IL-1β, IL-17A, MCP-1, TLR-2, TLR-4, CD11b, and other molecules involved in monocytes and platelets activation (like CCL2, MMP-1, MMP-9, CCR2, CCR1, IL-10, and S100A8) (9, 23, 34).

**Figure 9:**
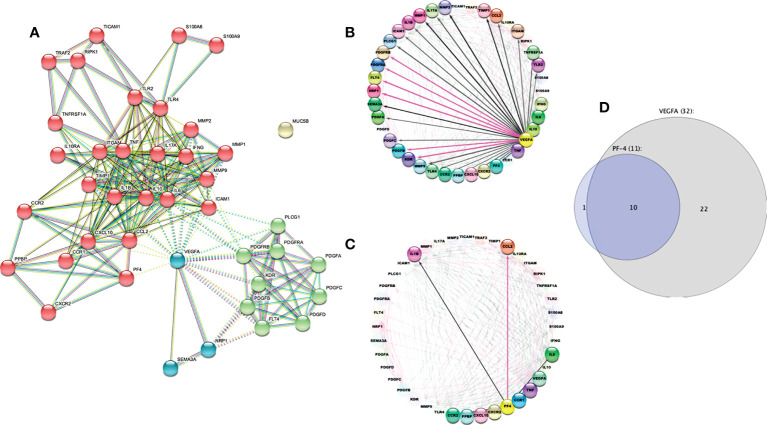
STRING protein–protein interaction diagram. **(A)** The STRING network shows protein-protein interactions. Different line colors represent the types of evidence for the association between proteins: experiments (pink line), databases (light blue line), co-expression (black line), text mining (green line) and “protein homology (violet line).” Based on the MCL clustering method (inflation parameter: 4). The solid and the dotted lines indicate connections within the same and different clusters, respectively. **(B)** The circular layout shows interactome to VEGF-A, and **(C)** shows interactome related to PF4. The black line indicates computational interaction, and the pink line indicates experimental interaction. **(D)** Venn Euler diagram showing the number of nodes in each logical grouping.

STRING analysis resulted in 41 nodes with an average node degree of 10.3 and a PPI enrichment p-value of less than 1.0e−16. Four main clusters were found using the MCL clustering method (similar to the results in **Figure 7B**). One of them was involved in inflammation (red bubbles). Two clusters were related to angiogenesis (green and blue bubbles), both mediated by VEGF-A. The last cluster was with MUC5B, which apparently had no relationship with the other evaluated molecules (**Figure 9A**). The findings were also supported by the REVIGO analysis (**Supplementary Excel file**).

We confirmed that VEGF-A is implicated in inflammation and angiogenesis (**Figure 9B**), whereas PF4 is related exclusively to the inflammatory process (**Figure 9C**) using Cytoscape analysis. This tool allowed us to predict the outcome of complex immune interactions between molecules associated with platelets and monocytes. In addition, 32 molecules were closely associated with VEGF-A, ten were involved in PF4 networks too, and only one molecule was related exclusively to PF4 (**Figure 9D**).

## Discussion

The role of platelets in the pathophysiology of TB has been recently explored; reports indicate that ATB patients have higher levels of platelets compared to LTB patients, and thrombocytosis is associated with greater severity and a worse prognosis for these patients (8, 24, 25). Platelets can mediate immunological mechanisms by forming aggregates with immune cells and releasing chemokines and growth factors (26).

In the context of TB, platelets mediate granuloma formation because the production of chemokines allows the recruitment of cells of the innate response such as neutrophils, monocytes, and macrophages. Moreover, platelets have the ability to induce a monocyte profile with strong collagenase activity, as well as to induce the differentiation of monocytes into multinucleated giant cells (9, 27).

In consonance with a previous report (8), we found that DS-TB patients had an elevated frequency of circulating monocytes and platelets. However, the frequency of monocytes’ subpopulations (classical and non-classical) was similar between LTB and DS-TB. The classical monocytes from DS-TB patients had an altered phenotype characterized by high expression of CD11b and TLR-2. Previously, it had been reported that P-selectin mediates the interaction of platelets with monocytes (7), and that the upregulation of CD11b is related to increased circulating leukocyte-platelet aggregates (28).

Platelets can be activated by directly recognizing mycobacteria through receptors such as TLR-2 and TLR-4, relevant molecules to induce the secretion of pro-inflammatory cytokines (7, 29). In this study, we found that TLR-2 was increased on classical monocytes in DS-TB patients, and these patients also displayed elevated levels of IL-1β, IL-6, and IP-10. Consequently, a pro-inflammatory environment was favored, and this condition probably intensified platelet activation. In this regard, reports have shown that pro-inflammatory cytokines enhance platelet production by triggering megakaryocytopoiesis (30).

Although we were unable to quantify IL-17A (samples were obtained from 2016 to 2018, and this cytokine has a rapid denaturation), elevated levels of IL-17A have been reported in ATB, and reports suggest that it induces the release of platelet factors (23, 35). Using the analysis STRING, we identified that VEGF-A interacts with IL-17A. In concordance with other reports indicating a loop, IL-17A might facilitate VEGF-A-mediated angiogenesis by enhancing VEGF-A production (36, 37). Furthermore, IL-17A stimulates the production of alarmins such as S100A8/A9, which mediates lung damage during TB; this alarmin activates platelets through an interaction with TLR-4 expressed on the platelet surface (34, 38). Thus, we speculate that the IL-17A pathway may participate in the platelet–classical monocyte-inflammation axis observed in this study during DS-TB.

IL-6 was increased in DS-TB patients, and evidence suggests that this cytokine promotes thrombocytosis and regulates thrombopoietin levels (39). Elevated PF4 levels (which are produced specifically by activated platelets) in DS-TB patients, compared to LTB, suggest that DS-TB patients have an increased presence of activated platelets, which in turn secrete factors to stimulate the inflammatory process. In consonance with another study (12), it was associated with radiological severity. Furthermore, PF4 mediates the migration of monocytes CCR1+ (22), a mechanism that could be involved during the Mtb infection to recruit monocytes and maintain the inflammatory process. Our data indicated a strong correlation between platelets, inflammatory cytokines and classical monocytes, whereas non-classical monocytes negatively correlated with platelet factors. We suggest that during DS-TB, platelets establish an inter-cellular communication with classical monocytes, probably those TLR-2+, that favors the inflammatory process and angiogenic factors that consequently lead to lung damage.

VEGF-A recently gained attention in the context of TB. On the one hand, it performs angiogenic mechanisms related to the spread of the bacteria, but on the other hand, it is also a chemokine for monocyte recruitment (14, 18). VEGF-A was increased in DS-TB patients and positively correlated with platelets and classical monocytes. The high level of VEGF-A can be attributed to the increase in monocytes and platelets, two of the main producers.

*In vitro* studies and murine models indicated that VEGF-A could promote platelet production by accelerating megakaryocyte maturation when interacting with its receptor, VEGFR1 (by paracrine or autocrine pathways). Platelets and monocytes can also produce this receptor (40, 41). Furthermore, VEGF-A promotes angiogenesis and regulates the expression of matrix metalloproteinases (MMP), leading to tissue destruction. Similarly, an excessive inflammatory response produces tissue damage and metalloproteinase expression, which could promote cavitation and fibrosis in the lung (15–17). Specifically, it has been described that VEGF-A can modulate the expression of MMP-1 and MMP-9, and MMP-9 can also regulate the expression of VEGF-A (15, 16, 42).

In addition, it is well known that the higher expression of extracellular MMP will lead to a higher degradation of lung tissue, and it is associated with pulmonary cavity formation in patients with ATB (17). The radiological data shown by our patients was consistent with that. The vast majority presented at least two cavitations; some had lung parenchyma destruction that had left them with sequelae of pulmonary fibrosis. The association of all these factors could explain why platelets are related to further clinical and radiological severity, the progression of the disease, and lower survival.

PF4 and VEGF-A levels have been associated with TB development. While VEGF-A can be considered a potent angiogenic mediator that favors pulmonary TB lesions, PF4 is related to the inflammatory process (8, 9, 14). We identified two clusters of TB patient groups through the PCA analysis; one group had increased, specifically, inflammatory factors, and the second group, angiogenic factors. Thus, in this study, we showed evidence that platelets are increased in DS-TB patients; moreover, these patients can be divided into groups with inflammatory or angiogenic factors, suggesting that PF4 and VEGF-A levels are determinants of the clinical status of DS-TB. Unfortunately, the bioinformatic analysis performed in our study did not identify a MUC5B-dependent network, even in DS-TB patients with extensive lung damage.

Each monocyte subpopulation has a specific phenotype and well-established functions. In this study, we provided evidence that the frequency of classical monocytes TLR-2+ was increased in DS-TB. These patients had positive correlations between classical monocytes and platelets, platelet-factor, and pro-inflammatory cytokines, and this phenotype is determinant for lung damage. Another report indicated that ATB patients have an expansion of non-classical monocytes compared to a negative tuberculin test group (43); this discrepancy with our study is probably because our TB patients are exclusively drug-sensitive. At least in a mouse model (44, 45), the classical monocytes have been identified as the main subpopulation that migrates to the site of infection to differentiate into macrophages or dendritic cells. However, studies in murine models have indicated that the retention of classic monocytes in the lung predisposes to lung damage (46, 47).

**Figure 10:**
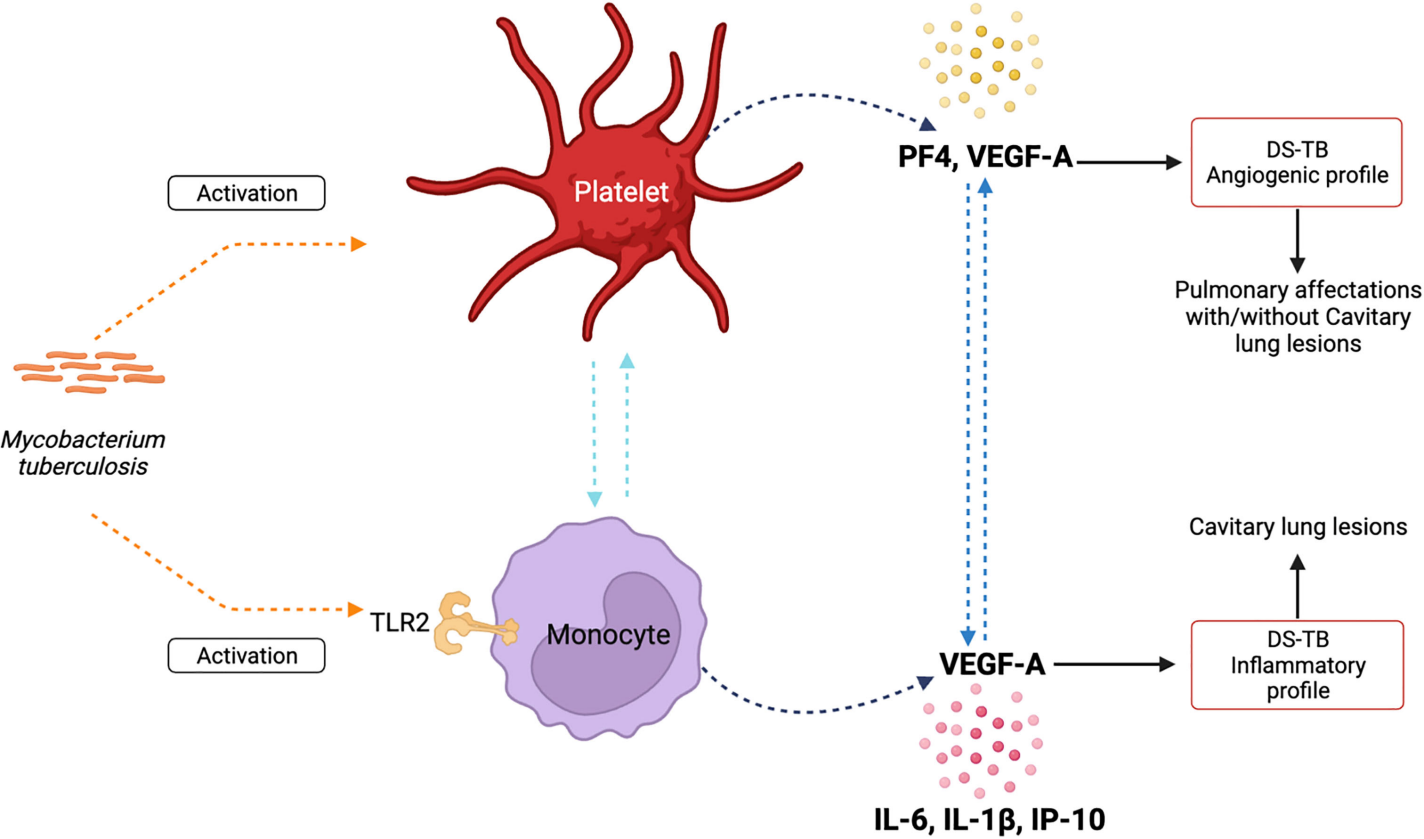
Summary of the presumptive relationship between platelet and classical monocytes to maintain an inflammatory environment and induce lung damage during active TB.

In summary, our data suggest that in DS-TB, Mtb induces activation of platelets and classical monocytes, probably through TLR-2. Platelets produce high levels of PF4, and both platelets and classical monocytes could be the main contributors to VEGF-A. Moreover, classical monocytes correlated positively with high levels of IL-1β, IL-6, and IP-10. Thus, platelets and classical monocytes could establish inter-cellular communication to promote the activation in both ways (**Figure 10**, left). According to angiogenic factors and pro-inflammatory profile, DS-TB patients can be divided into two groups: one group displays a strong angiogenic profile, and although these patients show pulmonary affections, not all patients develop cavitary lung lesions; and the second group has an inflammatory profile and 100% of patients show cavitation formation (**Figure 10**, right). All data suggest that platelets and classical monocytes (TLR-2+) during DS-TB enhance the inflammatory response, and consequently, lung damage is severe.

Thus, our study is not free of limitations. However, our study shows strong correlations, suggesting the existence of the platelet–classical monocytes–inflammation axis to mediate the lung damage during DS-TB. We did not confirm this interaction and still question whether TLR-2 is responsible for mediating the pro-inflammatory function in classical monocytes. Further, *in vitro* studies are necessary to clarify if platelets stimulate classical monocytes or if the latter is responsible for inducing the activation of platelets.

## Data availability statement

The original contributions presented in the study are included in the article/**Supplementary Material**. Further inquiries can be directed to the corresponding author.

## Ethics statement

The studies involving human participants were reviewed and approved by the Institutional Ethics and Scientific Committee. The patients/participants provided their written informed consent to participate in this study.

## Author contributions

Conceptualization, LC-G. Methodology, AU-S, JF-G, and LR-L. Validation, AC-L and LR-L. Formal analysis, AU-S, JF-G, GP-R, and LC-G. Investigation, AU-S. Resources, IB-R. Data curation, JF-G and GP-R. Writing—original draft preparation, AU-S and JF-G. Writing—review and editing, LC-G. All authors have read and agreed to the published version of the manuscript.

## Acknowledgments

We are grateful to the clinician staff of the Tuberculosis Clinic and Damaris Romero-Rodríguez from Flow Cytometry Core Facility, both at the Instituto Nacional de Enfermedades Respiratorias Ismael Cosio Villegas in Mexico City.

## Conflict of interest

The authors declare that the research was conducted in the absence of any commercial or financial relationships that could be construed as a potential conflict of interest.

## Publisher’s note

All claims expressed in this article are solely those of the authors and do not necessarily represent those of their affiliated organizations, or those of the publisher, the editors and the reviewers. Any product that may be evaluated in this article, or claim that may be made by its manufacturer, is not guaranteed or endorsed by the publisher.
