# Supplementary material for: High levels of PF4, VEGF-A, and classical monocytes correlate with the platelets count and inflammation during active tuberculosis
Source: Front Immunol. 2022 Oct 17;13:1016472. doi: 10.3389/fimmu.2022.1016472 (PMC9618821; doi:10.3389/fimmu.2022.1016472)
Supplement: Supplementary file 2 [file DataSheet_1.pdf]

Supplementary Material

High levels of PF4, VEGF-A, and classical monocytes correlate with the platelets count and inflammation during active tuberculosis

Alexia Urbán-Solano[1], Julio Flores-González[1], Alfredo Cruz-Lagunas[2], Gloria Pérez-Rubio[3], Ivette Buendia-Roldan[4], Lucero A. Ramón-Luing[1] and Leslie Chavez-Galan[1*]

[1]Laboratory of Integrative Immunology, Instituto Nacional de Enfermedades Respiratorias Ismael Cosío Villegas, Mexico City, Mexico

[2]Laboratory of Immunobiology and Genetic, Instituto Nacional de Enfermedades Respiratorias Ismael Cosío Villegas, Mexico City, Mexico

[3] HLA Laboratory, Instituto Nacional de Enfermedades Respiratorias Ismael Cosío Villegas, Mexico City, Mexico

[4] Translational Research Laboratory on Aging and Pulmonary Fibrosis, Instituto Nacional de Enfermedades Respiratorias Ismael Cosío Villegas, Mexico City, Mexico

*** Correspondence:**
Leslie Chávez-Galán
lchavezgalan@gmail.com; lchavez_galan@iner.gob.mx

Figures

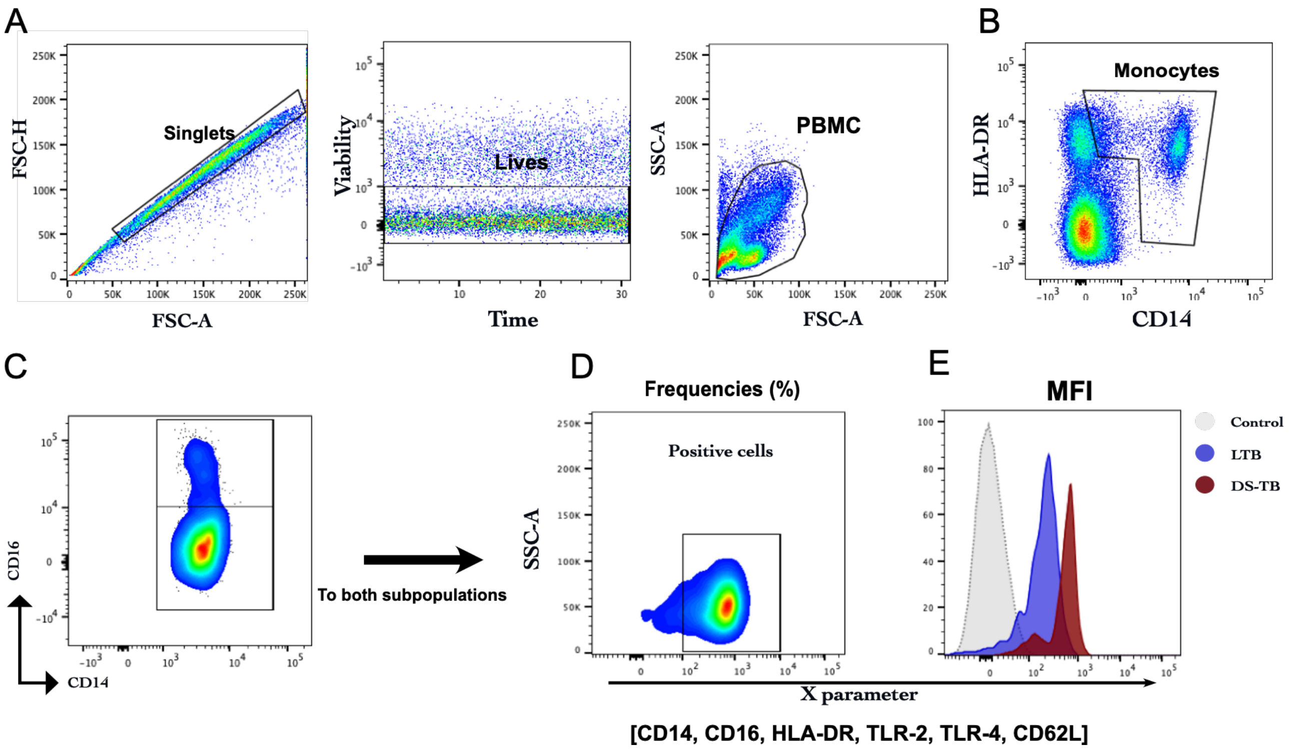

Figure S1. Gating strategies for flow cytometrical analysis. Representative example of the results obtained in cell subpopulation monocytes subsets from peripheral blood analysis (5,000 threshold): singlets cells **(A)** were identified through forward scatter (FSC-A versus FSC-H). Then, viable cells were selected, followed by forward scatter (FSC) and side scatter (SSC) dot plot to selected live monocytes based on HLA-DR and CD14 expression **(B)**. Two subpopulations of monocytes were identified based on CD14 and CD16 expression as Classical ($CD14^{+}$) and Non-classical ($CD14^{+}CD16^{+}$) **(C)**. The frequency **(D)** and mean fluorescent intensity (MFI) **(E)** of indicated molecules were calculated. All MFI analyzed included a latent (blue), active (red) tuberculosis patients and a negative control (gray).

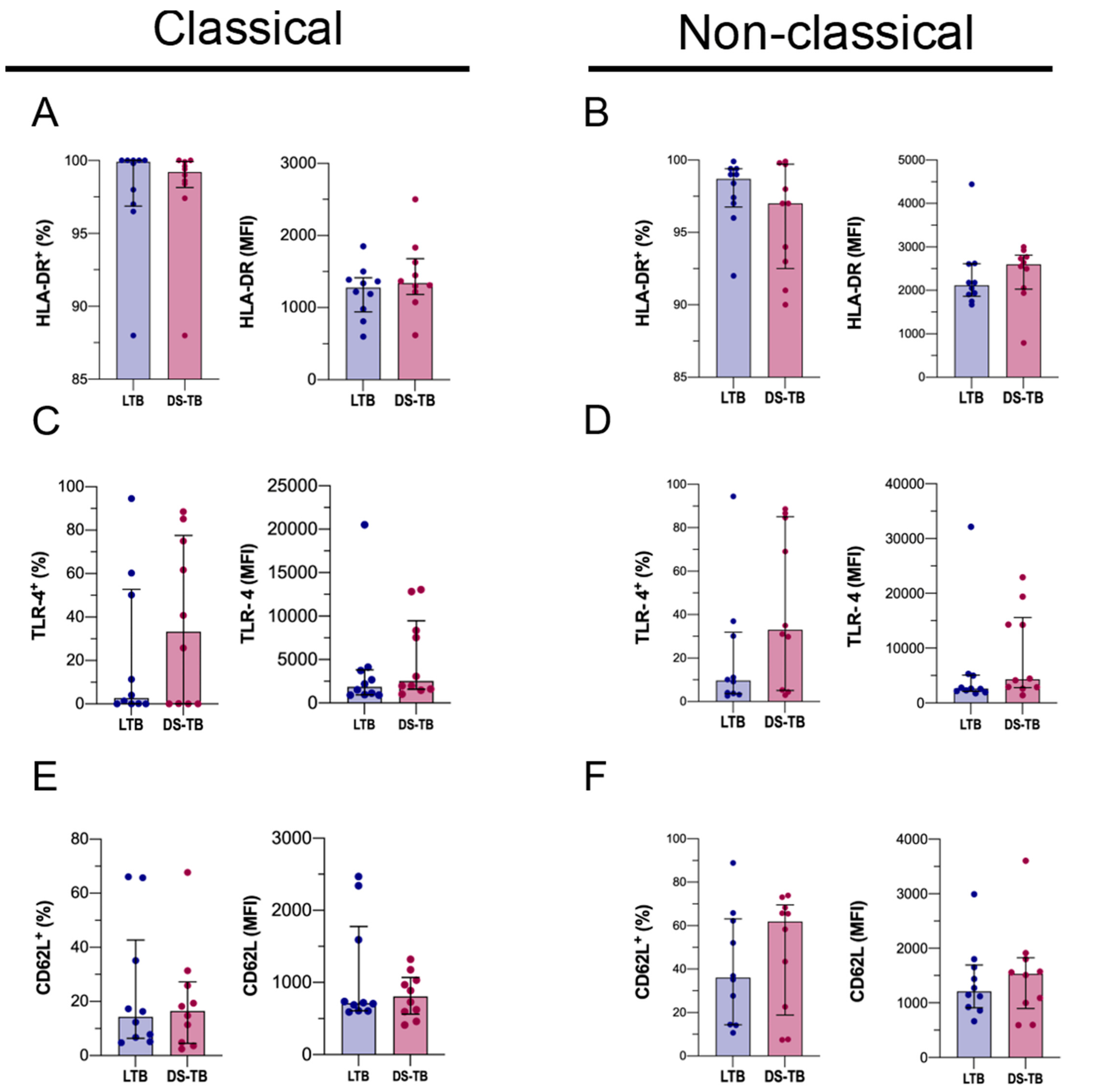

Figure S2. Frequency of subsets monocytes cells. Frequency and mean fluorescent intensity (MFI) of total monocytes cells in latent (LTB) and active (DS-TB) TB patients according to HLA-DR **(A, B),** TLR-4 **(C, D)** and CD62L **(E, F)** on classical (A, C and E) and non-classical (B, D and F) monocytes subsets. Data are shown as median with interquartile range (IQR, 25–75). The statistical comparison was performed using Mann-Whitney U Test.

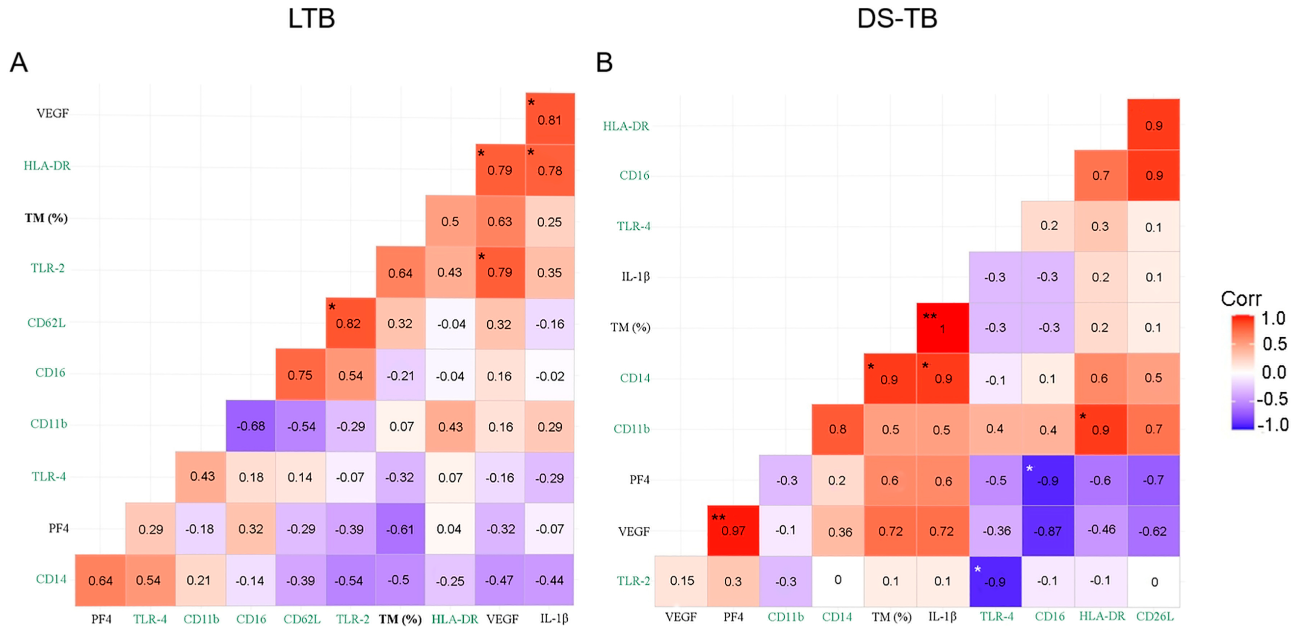

Figure S3. Correlation between Total monocytes (TM) and plasmatic levels of soluble molecules in latent (A) and active (B) TB patients. Correlations are presented with the value of Spearman's Rho in the corresponding box. Significant correlations are represented with asterisks (* $p < 0.05$, ** $p < 0.01$). The red color indicates a strong positive correlation and the blue color indicates a strong negative correlation. Green labels are mean fluorescent intensity values.

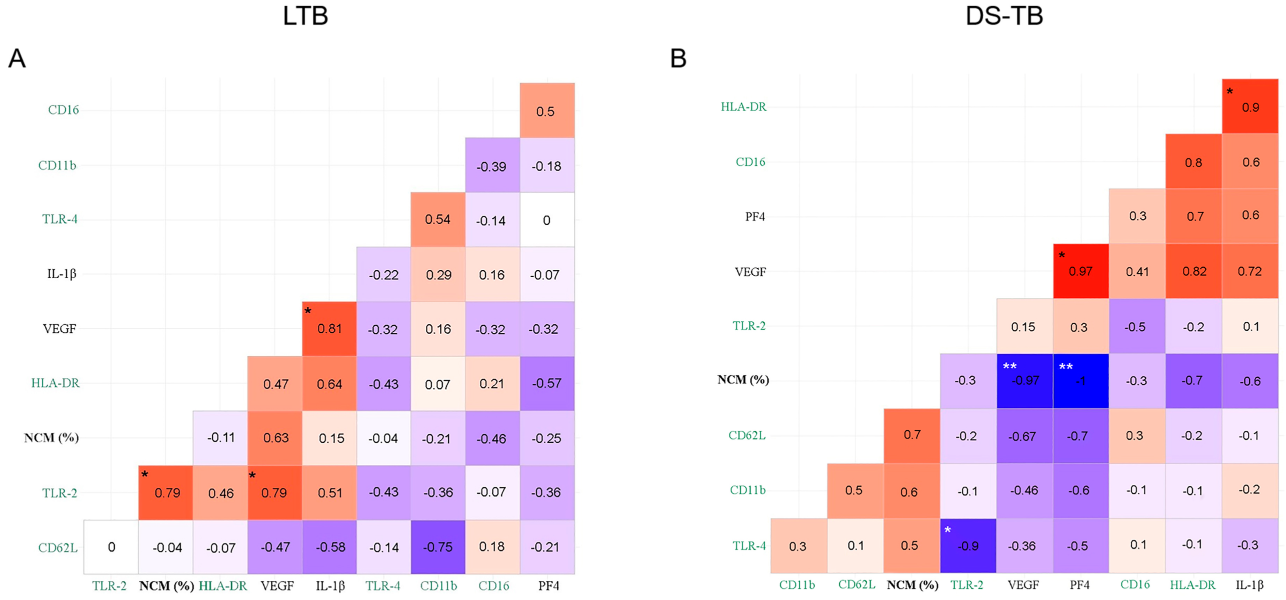

Figure S4. Correlation between non-classical monocytes (NCM) and plasmatic levels of soluble molecules in latent (A) and active (B) TB patients. Correlations are presented with the value of Spearman's Rho in the corresponding box. Significant correlations are represented with asterisks (* p

< 0.05, **$p < 0.01$). The red color indicates a strong positive correlation and the blue color indicates a strong negative correlation. Green labels are mean fluorescent intensity values.

Tables

Table S1. Antibodies used for flow cytometry, ELISA and Bio-Plex.

Molecule/Product	Conjugate to	Clone	Catalog	Company
CD16	PE-Cy7	3G8	302016	Biolegend
CD11b	PerCP-Cy5.5	ICRF44	301328	Biolegend
CD284	PE	HTA125	312806	Biolegend
CD282	FITC	TL2.1	309706	Biolegend
HLA-DR	APC-Cy7	L243	307617	Biolegend
CD14	BV510	63D3	367123	Biolegend
CD62L	BV421	DREG-56	304828	Biolegend
Viability	PE TexRed	NA[‡]	423109	Biolegend
CXCL4/PF4	NA[‡]	NA[‡]	DPF40	R&D System
MCP-1	NA[‡]	NA[‡]	438804	BioLegend
IL-17A	NA[‡]	NA[‡]	433914	Biolegend
MUC5B	NA[‡]	NA[‡]	MBS2024599	MyBioSource
IL-6	NA[‡]	NA[‡]	171B0006M	Bio-Rad Laboratories
IP-10	NA[‡]	NA[‡]	171B5020M	Bio-Rad Laboratories
VEGF-A	NA[‡]	NA[‡]	171B5027M	Bio-Rad Laboratories

PDGF-AB	NA‡	NA‡	171B5024M	Bio-Rad Laboratories
Reagent Kit III with Flat Bottom Plate	NA‡	NA‡	171-304090M	Bio-Rad Laboratories
Human Cytokine Standards	NA‡	NA‡	171D50001	Bio-Rad Laboratories

‡NA, not applicable.

Table S2. Comparison of parameters evaluated with the healthy donors group.

Biomolecule	HD	LTB	DS-TB	*p* value HD vs LTB	*p* value HD vs DS-TB
PF4 (ng/mL)	2250 (1929-2816)	2012 (1213-2727)	3108 (2578-3750)	ns	ns
PDGF-BB (pg/mL)	420.2 (30.81-635.9)	65.98 (28.46-199.1)	186 (19.39-257.9)	ns	ns
VEGF-A (pg/mL)	0 (0-0)	0 (0-0.55)	9.64 (0-22.69)	ns	0.0009
MCP-1 (pg/mL)	79.11 (61.22-94.48)	99.04 (88.15-118.3)	91.16 (71.91-123.0)	ns	ns
IL-6 (pg/mL)	0.22 (0-	0.73 (0.4425-	14.78 (4.428-	ns	0.0002

	1.275)	1.0)	31.60)		
IL-1β (pg/mL)	0 (0-0)	0.035 (0-0.1425)	0.465 (0.33-0.7625)	ns	<0.0001
IP-10 (pg/mL)	118.3 (103.7-332.7)	282 (161.7-473.6)	2718 (1033-5658)	ns	0.0003

Data are shown as median with interquartile range (IQR, 25–75). The statistical comparison was performed using Mann-Whitney U Test. HD: healthy donor, LTB: latent tuberculosis, DS-TB: drug-sensitive tuberculosis, PF4: platelet factor-4, PDGF-BB: platelet-derived growing factor, VEGF-A: vascular endothelial growing factor, MCP-1: monocyte chemoattractant protein-1, IL-6: interleukin-6, IL-1b: interleukin-1 beta, IP-10: Interferon-γ inducible protein 10, ns: not significant.
